# Killed *Propionibacterium acnes* enhances immunogenicity and tumor growth control of a dendritic-tumor cell hybrid vaccine in a murine melanoma model

**DOI:** 10.1371/journal.pone.0205148

**Published:** 2018-10-09

**Authors:** Mayari E. Ishimura, Daniela Teixeira, Gabriela da P. Silveira, Mônica Gambero, Gabriel A. C. Gama, Bruna S. O. Pimenta, Elaine G. Rodrigues, Ieda M. Longo-Maugéri

**Affiliations:** 1 Disciplina de Imunologia, Departamento de Microbiologia, Imunologia e Parasitologia, Universidade Federal de São Paulo-Escola Paulista de Medicina, São Paulo, Brazil; 2 Laboratório de Imunobiologia do Cancer, Disciplina de Biologia Celular, Departamento de Microbiologia, Imunologia e Parasitologia, Universidade Federal de São Paulo-Escola Paulista de Medicina, São Paulo, Brazil; Mie University Graduate School of Medicine, JAPAN

## Abstract

Hybrid vaccines have been investigated in clinical and experimental studies once expresses total antigens of a tumor cell combined with the ability of a dendritic cell (DC) to stimulate immune responses. However, the response triggered by these vaccines is often weak, requiring the use of adjuvants to increase vaccine immunogenicity. Killed *Propionibacterium acnes* (*P*. *acnes*) exerts immunomodulatory effects by increasing the phagocytic and tumoricidal activities of macrophages, promoting DC maturation, inducing pro-inflammatory cytokines production and increasing the humoral response to different antigens. Here, we evaluated the effect of *P*. *acnes* on a specific antitumor immune response elicited by a hybrid vaccine in a mouse melanoma model. Hybrid vaccine associated with *P*. *acnes* increased the absolute number of memory T cells, the IFN-γ secretion by these cells and the IgG-specific titers to B16F10 antigens, polarizing the immune response to a T helper 1 pattern. Furthermore, the addition of *P*. *acnes* to a hybrid vaccine increased the cytotoxic activity of splenocytes toward B16F10 *in vitro* and avoided late tumor progression in a pulmonary colonization model. These results revealed the adjuvant effect of a killed *P*. *acnes* suspension, as it improved specific humoral and cellular immune responses elicited by DC-tumor cell hybrid vaccines.

## Introduction

Dendritic cells (DC) are antigen-presenting cells (APCs) that process and express tumor antigens using the major histocompatibility complex (MHC) class I and II molecules, playing a central role in the induction of T cell immunity. Therefore, DC vaccines are an important cancer immunotherapy strategy that elicits direct immune responses and activates *naïve* lymphocytes to target specific tumor antigens. Indeed, based on many clinical and experimental studies, vaccination with DCs pulsed with tumor lysate cells [1–3] or immunogenic peptides [4], DCs transfected with cDNAs of tumor antigens [5] and DC-tumor cell hybrid vaccines [6, 7] is safe and induces a T cell response, engendering tumor immunity. Nonetheless, the immune response triggered by these vaccines in clinical studies is often weak, necessitating the evaluation of an adjuvant to improve their immunogenicity.

*Propionibacterium acnes* (*P*. *acnes*), a gram-positive bacillus present in human skin microbiota [8–10], induces immunomodulatory effects on innate and adaptive immune responses when used as phenol- or heat-killed suspension. *P*. *acnes* treatment increases the phagocytic activity of macrophages and animal resistance after challenge with different pathogens, such as *Trypanosoma cruzi*, *Mycobacterium lepraemurium* and *Leishmania major* [11–15]. These effects were correlated with increased survival and a reduced number of parasites in *P*. *acnes*-treated animals compared to control groups.

Macrophages stimulated with *P*. *acnes in vitro* or obtained from *P*. *acnes*-treated animals also showed enhanced cytotoxic activity toward different tumor cells [16–18]. This observation corroborates the antitumor activity of *P*. *acnes* in experimental studies *in vivo* and in clinical trials when this bacterium was used simultaneously with chemotherapy/radiotherapy [12,19–22].

Despite the number of biological effects attributed to *P*. *acnes*, the mechanisms by which *P*. *acnes* modulates the immune system have only recently been clarified. *P*. *acnes* promotes the synthesis of pro-inflammatory cytokines, such as IFN-γ, IL-1, IL-6, TNF-α, IL-12 and IL-18 [23–25].

Because *P*. *acnes* induces these cytokines synthesis, it was considered a T helper 1 (Th1) antigen. However, as shown in our previous studies, this bacterium exacerbates the Th2 response to ovalbumin (OVA) when injected simultaneously with this antigen in mice. Nevertheless, a *P*. *acnes* suspension changed the typical Th2 immune response to a Th1 pattern when animals were sensitized after treatment with *P*. *acnes*. In addition, *P*. *acnes* modulates the cellular immune response through a direct action on APCs, [26–28].

The addition of *P*. *acnes* to bone marrow cell cultures increases the expression of CD11c, MHCII and costimulatory molecules on the surface of DCs [29]. Moreover, intravenous or intraperitoneal injections of *P*. *acnes* in animals increase the number of DCs in circulation or in the peritoneal cavity, respectively [18, 30]. Moreover, the subcutaneous injection of *P*. *acnes* increases the absolute number of DCs in the bone marrow of treated animals, and in culture, these cells show increased expression of both CD11c and MHCII molecules, cytokine synthesis and the ability to present antigens to T lymphocytes. Therefore, *P*. *acnes* acts on DCs, inducing their recruitment, activation and maturation [31].

This *P*. *acnes*-mediated modulation of APCs, particularly DCs, could explain the adjuvant effect induced by this bacterium in a plasmid DNA *Trypanosoma cruzi* vaccine, which increased the *T*. *cruzi* antigen-specific Th1 immune response by increasing IFN-γ synthesis by CD4 T cells and reducing the IgG1/IgG2a ratio [32]. Other studies have also observed a *P*. *acnes*-dependent modulation of the antibody response in different experimental models, as this bacterium increases the titers of specific antibodies and modifies the polarization of this response [27, 33].

Because *P*. *acnes* has been shown to modulate humoral and cellular immunity, the aim of the present study was to evaluate the ability of this adjuvant to improve the specific antitumor immune response induced by hybrid vaccines in a murine melanoma model. This vaccine was selected because hybrid vaccines unite the ability of DCs to elicit an immune response with the total repertoire of the tumor antigens, including unidentified antigens, presented by both MHC class I and II pathways.

Here, the use of *P*. *acnes* as adjuvant for a hybrid tumor vaccine polarizes humoral and cellular immune responses to a Th1 pattern, enhances the cytotoxic activity of splenocytes toward tumor cells and increases the absolute number of CD4 and CD8 memory T cells. Most importantly, these findings corroborate our *in vivo* experiments, showing that hybrid vaccines with the *P*. *acnes* adjuvant inhibited tumor progression.

## Materials and methods

### Animals

Inbred female 8-week-old C57Bl/6j mice purchased from the Center for Development of Experimental Models (CEDEME) at Universidade Federal de São Paulo (UNIFESP) were housed in standard cages and maintained on a 12 hour light/dark cycle at a controlled temperature with water and food available *ad libitum*. Animals were monitored every 2 or 3 days and none of them received analgesics or anesthetics. None animal died without euthanasia which was performed in a CO_2_ chamber. This study was carried out in strict accordance with the recommendations in the Guide for the Care and Use of Laboratory Animals of the Brazilian National Council of Animal Experimentation (http://www.cobea.org.br). The protocols were approved by the Ethics Committee on the Use of Laboratory Animals from Universidade Federal de São Paulo (0120/12).

### Adjuvant

A heat-killed *P*. *acnes* suspension was prepared using the method reported by Squaiella *et al*. (2006) [31].

### Tumor cells

The B16F10 murine melanoma cell line, obtained from the Cell Bank of Rio de Janeiro (BCRJ), was cultured (37°C, 5% CO_2_) in complete medium (CM) comprising RPMI-1640 (Gibco-31800022) supplemented with 10% fetal bovine serum (Gibco-12657), 2 mM L-glutamine (Gibco-25030081), 1 mM sodium pyruvate (Gibco-11360070), a 1% vitamin solution (Gibco-11120052), 1% nonessential amino acids (Gibco-11140050), 10,000 units of penicillin/10,000 μg of streptomycin (Gibco-15140122), 28 mM HEPES (Gibco-11344041), 23.8 mM sodium bicarbonate (Nuclear-311894) and 55 mM 2-mercaptoethanol (Gibco-21985023). B16F10 cells were used for *in vivo* experiments or to obtain tumor antigens. B16F10 cells from culture flasks or isolated from tumor-bearing mice *in vivo* were used to recover tumor antigens from the total lysate through repeated freeze-thaw cycles in liquid nitrogen and a 37°C water bath, respectively. The protein concentration was determined using the Bradford method [34].

### DC generation

Mouse bone marrow precursors cells were obtained on day 0, and 2.5x10^5^ cells/mL were cultured in high glucose Dulbecco’s Modified Eagle’s Medium (DMEM) (Gibco-12100046) supplemented with 20 ng of GM-CSF/mL (Gibco-PMC2013), 10% fetal bovine serum (Gibco-12657), 1 mM sodium pyruvate (Gibco-11360070), a 1% vitamin solution (Gibco-11120052), 1% nonessential amino acids (Gibco-11140050), 10,000 units of penicillin/10,000 μg of streptomycin (Gibco-15140122), 44 mM sodium bicarbonate (Nuclear-311894) and 55 mM 2-mercaptoethanol (Gibco-21985023). On days 4 and 7, medium (1 mL) was added to the culture, and medium without GM-CSF was added only on day 7. On day 9, the cells were collected and the cell number was determined.

### Fusion of dendritic and tumor cells

Irradiated (200 Gy) B16F10 cells were fused with DCs at the same proportion (1:1) using polyethylene glycol (PEG) (Sigma-P7306). Subsequently, the cells were maintained for 1 hour at 37°C and 5% CO_2_. The cells were collected and the cell number was determined. DCs and B16F10 cells were stained with Cell Trace Violet (Life Technologies-C34571) and CFSE (Invitrogen-V12883), respectively, prior to fusion to investigate the fusion efficiency. The cells were analyzed using flow cytometry (Attune Acoustic Focusing Cytometer–Life Technologies, USA), and this fusion protocol was verified to generate approximately 20% hybrid cells.

### Treatment protocol

The mice were subcutaneously (sc) injected with two doses of 5x10^5^ hybrid cells with or without *P*. *acnes* (140 μg protein/animal/dose). The interval between doses was 2 weeks. Controls groups received PBS or 140 μg of *P*. *acnes* protein/dose.

### Detection of specific antibody responses against tumor cells

A standardized Enzyme Linked Immunosorbent Assay (ELISA) was used to detect specific antibodies against tumor antigens. Plates (Costar-3590) were coated with B16F10 total lysate (50 μg protein/well) from cells that had been expanded *in vitro* (as described above) in carbonate/bicarbonate buffer. After an overnight incubation (4°C), the plates were washed three times with PBS/0.05% Tween 20 (Synth) (PBST), and the wells were blocked with PBST/5% skim powdered milk/1% bovine serum albumin (BSA) (Sigma-A7906) for 1 hour at room temperature (RT). The plates were washed again, and the samples (pooled sera from three mice/group) were diluted from 1:200–1:12,800 to detect total IgG or 1:50–1:6,400 to detect IgG1, IgG2a, IgG2b or IgG2c (100 μL/well). After a 2 hour incubation at RT, the plates were washed and incubated with 100 μL/well of horseradish peroxidase (HRP)-conjugated anti-mouse IgG (1:1,000) (Sigma-A5906), IgG1 (1:4,000) (Southern Biotech-107005), IgG2a (1:4,000) (Southern Biotech-108005) or IgG2c (1:4,000) (Southern Biotech-107905) for 1 hour (RT). Biotin anti-mouse IgG2b (1:4,000) (BD Pharmingen-550333) was also incubated for 1 hour, the plate was washed and incubated with streptavidin-peroxidase (eBioscience-184100) for 30 minutes at RT. After washing, peroxidase activity was assessed with 1 mg/mL of o-phenylenediamine (OPD, Sigma-P1526) and 0.03% of hydrogen peroxide (100 μL/well), and the reactions were terminated with 50 μL of 4N H_2_SO_4_ (Dinamica-13081) per well. The optical density was measured at 492 nm, and antibody titers were determined. Each titer corresponds to the first dilution of sera with absorbance values higher than 0.1. The antibody ratio was calculated by dividing the IgG1 titers by the IgG2c titers.

### Memory phenotype evaluation

Inguinal lymph nodes from control and vaccinated groups were obtained 24 hours after the injection of the second vaccine dose. Cellular viability and the absolute number of cells were determined by counting cells stained with Trypan Blue (Gibco-15250061). The cells were then incubated with normal mouse serum to block Fc receptors (30 minutes, 4°C). After washing, the cells were labeled with the following fluorochrome-conjugated anti-mouse monoclonal antibodies: anti-CD3FITC (BD-553062), anti-CD4APC (eBioscience-17004283), anti-CD8APC (eBioscience-17008183), anti-CD44PerCP-Cy5.5 (eBioscience-45044182), anti-CD62LPE (eBioscience-12062182) and anti-CD69 biotin (Invitrogen-HM40153). After a 30 minute incubation at 4°C, the cells were washed, labeled with Pacific Orange-conjugated streptavidin (Invitrogen-S32365) under the same conditions and analyzed using flow cytometry (BD FACSCanto II, USA) to determine the percentages of *naïve* (CD44^low^CD62L^high^), effector memory (EM = CD44^high^CD62L^low^) and central memory (CM = CD44^high^CD62L^high^) CD4 and CD8 T cells. The expression of the CD69 molecule was evaluated to measure cell activation. The absolute number of cells in each population was calculated by multiplying the total cell number by the percentages of each subset.

Memory phenotype was also analyzed in tumor infiltrating cells. Animals (n = 3) were intravenously (iv.) challenged with 3x10^5^ B16F10 cells seven days after the last immunization. Twenty days later, the mice were euthanized and lungs were extracted, cell suspension were obtained and tumor infiltrating lymphocytes were enriched using Percoll (GE Healthcare) gradient. After to block Fc receptors, the cells were washed and incubated with the following anti-mouse monoclonal antibodies under the same conditions above: anti-CD3PECy7 (BD Pharmingen-560591), anti-CD4FITC (eBioscience-11004182), anti-CD8APCCy7 (BD Bioscience-557654), anti-CD44APC (eBioscience-17044181), anti-CD45Pacific Blue (BioLegend-103126), anti-CD62LPE (eBioscience-12062181) and anti-CD69PECy5 (eBioscience-15069181). After washing, the cells were analyzed using flow cytometry (BD LSRFortessa, USA) to determine the percentages of *naïve*, effector memory, central memory and activated CD4 and CD8 T cells inside the population expressing CD45 on cell surface.

### Cytokine production by primed T lymphocytes *in vitro*

Spleen cells from control and vaccinated groups were obtained 14 days after the last vaccine dose and the cell number was determined. After to block Fc receptors, the cells were washed and incubated with the following anti-mouse monoclonal antibodies under the same conditions: anti-CD3APC (eBioscience-17003183), anti-CD4PerCP-Cy5.5 (eBioscience-45004282) and anti-CD8PE (eBioscience-12008183). After washing, the cells were analyzed using flow cytometry, and CD4 and CD8 T cells were sorted (BD FACSAria II–Cell Sorter, USA). Subsequently, 1x10^5^ cells from each T subpopulation were cultured with 2.5x10^4^ DCs and tumor antigens from total lysates of B16F10 cells isolated *in vivo*. After 5 days of co-culture (37°C, 5% CO_2_), supernatants were collected and stored at -20°C. The levels of IL-2, IL-4, IL-6, IL-10, TNF-α, IFN-γ and IL-17 were detected using the Th1/Th2/Th17 CBA kit (BD-560485) according to manufacturer’s instructions and flow cytometry (BD FACSCanto II, USA) to investigate the cytokine profiles.

### Cytotoxicity assay

B16F10 cells were cultured in complete medium containing [methyl-^3^H] thymidine (5μCi/mL) (Amersham Biosciences) for 24 hours at 37°C and 5% CO_2_. The cells were collected, washed and the cell number and viability were determined by counting cells stained with Trypan Blue (Gibco-15250061). 1x10^4^ B16F10 cells were co-cultured with splenocytes from the vaccinated or control groups at a ratio of 1 target cell to 50 effector cells, to detect cytotoxicity toward melanoma (E). Spontaneous lysis (S) was determined by culturing B16F10 cells alone in complete medium. After a 3.5 hour incubation (37°C, 5% CO_2_), the cells were harvested (PerkinElmer), and radioactivity was measured using a β-counter (MicroBeta^2^LumiJET-PerkinElmer). The percentage of lysed cells was determined by:

%Lysis=[(Scpm−Ecpm)Scpm]×100

### Tumor growth in controls or immunized groups

Animals were intravenously (iv.) challenged with 3x10^5^ B16F10 cells seven days after the last immunization. Fourteen or twenty days later, the mice were euthanized, the lungs were extracted and the number of nodules was determined. When we could not count the lung nodules due to intense colonization, this parameter was plotted on the graphs as 500 nodules. None animal showed severe signs of illness following tumor formation or adverse outcomes.

### Statistical analysis

The significance of the differences between the control and vaccinated groups was analyzed using one-way ANOVA followed by Tukey's Multiple Comparison Test using GraphPad Prism software version 5.0 (GraphPad Software, CA, USA). Differences were considered statistically significant at p<0.05.

## Results

### Hybrid vaccination induced a specific antibody response against melanoma antigens

**Fig 1:**
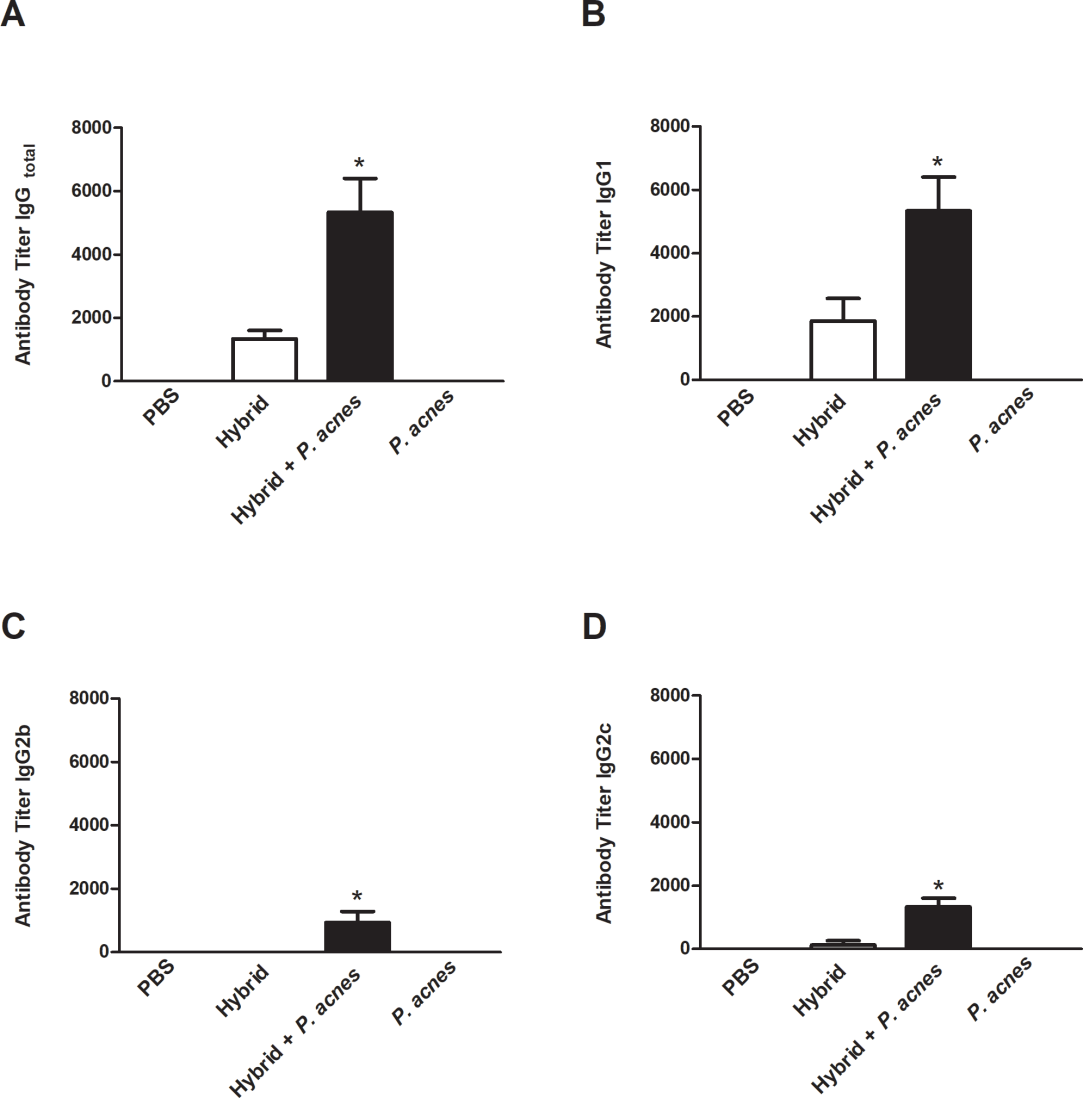
*P*. *acnes* increased the specific antibody response to melanoma antigens. C57Bl/6 mice (n = 3) were sc immunized with the hybrid vaccine in the presence or absence of *P*. *acnes* on days 0 and 14. Mice in the control groups received PBS or *P*. *acnes* only. Pooled sera were obtained on day 21, and the titers of specific IgG, IgG1, IgG2b and IgG2c antibodies to B16F10 antigens were determined using ELISAs. Each titer corresponds to the first dilution of sera that achieved an absorbance value higher than 0.1. The graphs present the mean ± SEM of three independent experiments. ANOVA with Tukey’s post-test *p<0.05, **p<0.01, ***p<0.001.

The hybrid vaccine induced the synthesis of low levels of IgG specific to melanoma antigens, including isotypes IgG1 and IgG2c. The addition of *P*. *acnes* to the hybrid vaccine increased the total IgG, IgG1, IgG2b and IgG2c titers specific to B16F10 antigens compared to immunization with the hybrid vaccine (p<0.05). As expected IgG2a was not detected in C57Bl/6 mice (data not show). Antibodies against melanoma antigens were not also detected in the PBS and *P*. *acnes* controls groups (Fig 1).

The IgG1/IgG2c ratio was calculated to analyze the balance between Th1 and Th2 patterns. In the Hybrid group, this ratio was 14.0, whereas this ratio was 4.0 in the Hybrid+*P*. *acnes* group (Table 1).

**Table 1:** *P*. *acnes* polarized the antibody response induced by the hybrid vaccine to a Th1 pattern.

Group	IgG subclasses titer		IgG1/IgG2c ratio
	IgG1	IgG2c	
**Hybrid**	1,866	133	14.0
**Hybrid + *P*. *acnes***	5,333	1,333	4.0

Serial dilutions of pooled sera from C57Bl/6 immunized mice (n = 3) were analyzed using ELISAs to detect the titers of IgG isotypes specific for B16F10 antigens. The mean IgG1 and IgG2c titers were calculated using the data from three independent experiments. The isotype ratio was obtained by dividing the mean IgG1 titer by the mean IgG2c titer.

### *P*. *acnes* addition to the hybrid vaccine increased the absolute numbers of memory T cell populations

**Fig 2:**
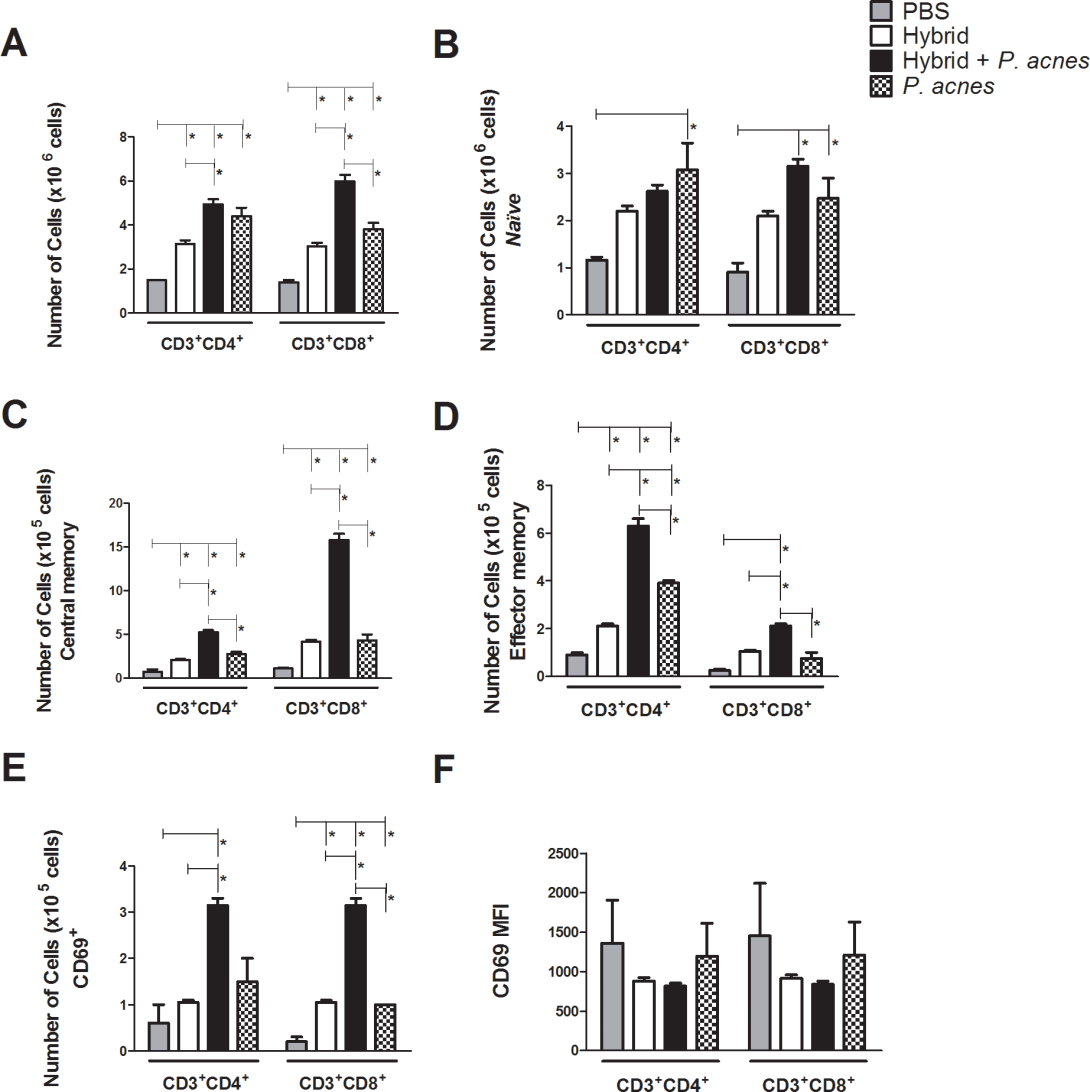
The addition of *P*. *acnes* to the hybrid vaccine increased the absolute numbers of memory and activated CD4 and CD8 T cells in the lymph nodes of immunized mice. Cells were collected from the inguinal lymph nodes of C57Bl/6 mice (n = 3) 24 hours after the injection of the second vaccine dose, and the total cell numbers were determined. Subsequently, the cells were stained with fluorochrome-conjugated monoclonal antibodies and analyzed using flow cytometry. The absolute number of cells in each population was calculated by multiplying the total cell number by the percentages of cells in each subset. The mean ± SEM absolute numbers of CD4 and CD8 T cells (CD3^+^CD4^+^ and CD3^+^CD8^+^) (A) and subpopulations of *naïve* (CD44^low^CD62L^high^) (B), CM (CD44^high^CD62L^high^) (C) and EM cells (CD44^high^CD62L^low^) (D) are presented in the graphs. The absolute numbers of activated CD4 and CD8 T cells (CD69^+^) (E) and their degree of activation based on CD69 mean fluorescence intensity (MFI) (F) were also investigated. The data shown in the graphs are representative of two independent experiments. ANOVA with Tukey’s post-test *p<0.05, **p<0.01, ***p<0.001.

Cells were collected from the draining lymph nodes and the memory phenotype was evaluated (S1 Fig). All treatments increased the absolute numbers of CD4 and CD8 T cells compared to PBS (Fig 2A).

The analysis of the absolute *naïve* T cell numbers showed increased numbers of CD8 T cells in the Hybrid+*P*. *acnes* and *P*. *acnes* groups and an increased number of CD4 T cells in the *P*. *acnes* group only (Fig 2B).

The Hybrid+*P*. *acnes* group exhibited a significant increase in the absolute numbers of central (CM) and effector memory (EM) CD4 T cells compared to the Hybrid and PBS groups (CM = 5.25x10^5^±0.25 and EM = 6.30x10^5^±0.30; CM and EM = 2.10x10^5^±0.10; CM = 0.75x10^5^±0.25 and EM = 0.90x10^5^±0.10, respectively) (Fig 2C and 2D).

Moreover the coadministration of the adjuvant and vaccine increased the absolute number of activated CD4 T cells (3.15x10^5^±0.15) compared to the PBS (0.60x10^5^±0.40) and Hybrid (1.05x10^5^±0.05) groups (Fig 2E), but did not alter the activation of these cells (Fig 2F). The administration of *P*. *acnes* alone increased the absolute numbers of CM (2.75x10^5^±0.25) (Fig 2C) and EM (3.90x10^5^±0.10) CD4 T cells (Fig 2D) compared to the PBS group. Nevertheless, the absolute numbers of CM and EM CD4 T cells in the *P*. *acnes* group were less than the numbers observed in the Hybrid+*P*. *acnes* group (Fig 2C and 2D).

Concerning the CD8 T cell population, the addition of *P*. *acnes* to the hybrid vaccine increased the number of CM T cells (15.75x10^5^±0.75) compared to the other groups (Hybrid: 4.20x10^5^±0.20; *P*. *acnes*: 4.35x10^5^±0.65 and PBS: 1.10x10^5^±0.10) (Fig 2C) and also increased the absolute numbers of activated cells (Hybrid+*P*. *acnes*: 3.15x10^5^±0.15; Hybrid: 1.05x10^5^±0.05; *P*. *acnes*: 1.00x10^5^±0.00; PBS: 0.20x10^5^±0.10) (Fig 2E), but no treatment modified the activation of this cell population (Fig 2F). Moreover, only the Hybrid+*P*. *acnes* group (2.10x10^5^±0.10) increased the absolute number of EM CD8 T compared with the Hybrid (1.05x10^5^±0.05), PBS (0.25x10^5^±0.05) and *P*. *acnes* (0.75x10^5^±0.25) groups (Fig 2D).

**Fig 3:**
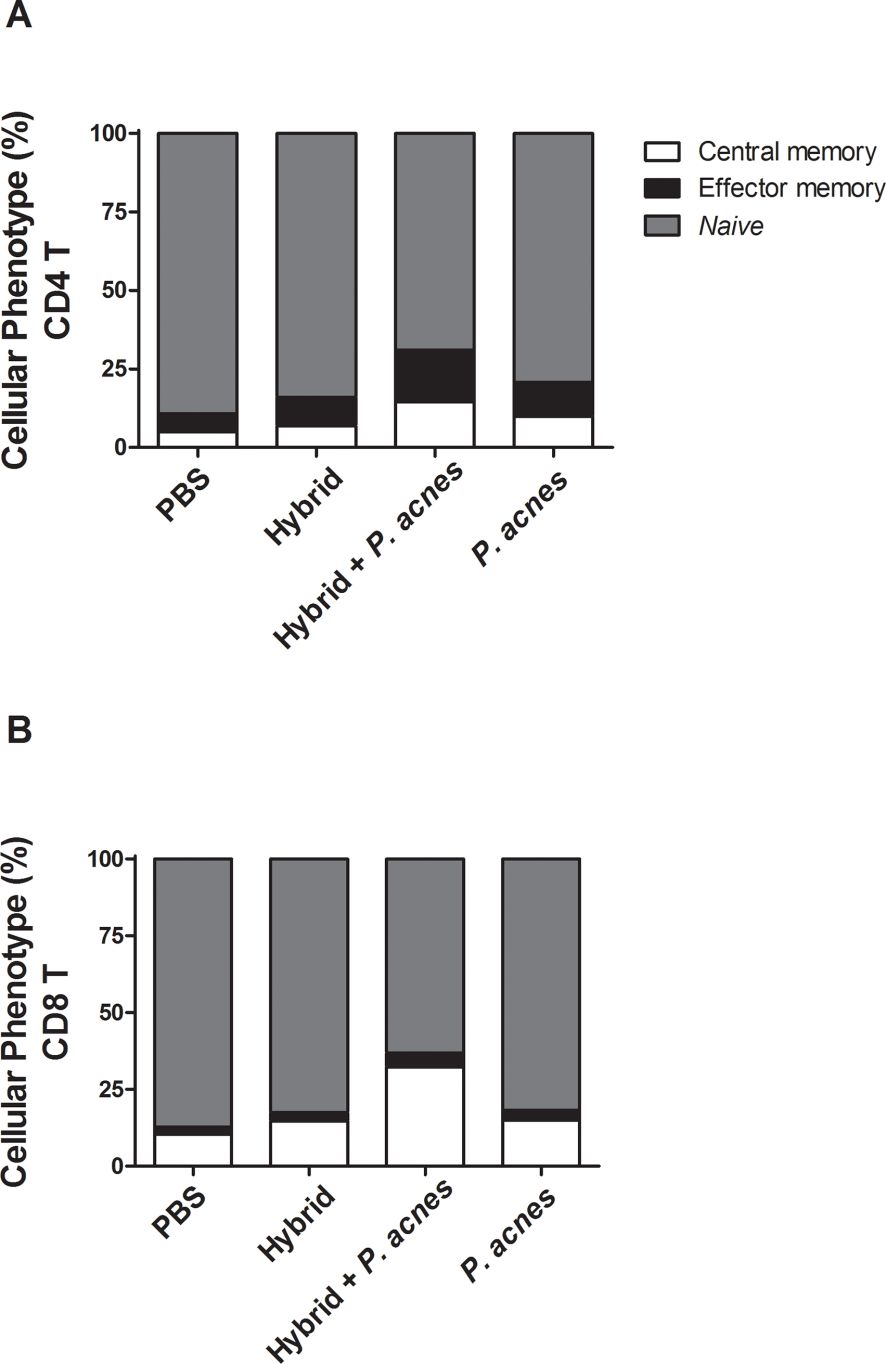
The addition of *P*. *acnes* to the hybrid vaccine increased the proportion of CM and EM CD4 and CD8 T cells. Cells were collected from the inguinal lymph nodes of C57Bl/6 mice (n = 3) 24 hours after the injection of the second vaccine dose and then stained with fluorochrome-conjugated monoclonal antibodies and analyzed using flow cytometry. The proportion of *naïve* (gray bar), CM (white bar) and EM (black bar) cells was calculated dividing the absolute number (mean) of each subset by the number obtained with the sum of *naïve*, CM and EM CD4 T (A) or CD8 T (B) absolute numbers (mean), then this quotient was multiplied by 100. The data shown in the graphs are representative of two independent experiments.

Moreover, when the proportion of each subset was calculated in relation to the total percentage of CM, EM and *naïve* CD4 T or CD8 T cells (Fig 3), the percentage of CM CD4 T cells was increased 2.9-fold, 2.1-fold and 1.5-fold in the Hybrid+*P*. *acnes* group compared with the PBS, Hybrid and *P*. *acnes* groups, respectively. For CD8 T cells, these values were increased 3-fold in the Hybrid+*P*. *acnes* group compared with the PBS group and 2.2-fold compared with the Hybrid and *P*. *acnes* groups. The number of EM CD4 T cells was increased 2.8-fold, 1.8-fold and 1.5-fold in the Hybrid+*P*. *acnes* group compared to the PBS, Hybrid and *P*. *acnes* groups, respectively. The percentage of EM CD8 T cells in the Hybrid+*P*. *acnes* group was increased 2-fold, 1.7-fold and 1.4-fold compared with the PBS, Hybrid and *P*. *acnes* groups, respectively (Fig 3).

The memory phenotype was also investigated in tumor infiltrating cells (S2 Fig). However, it was not observed difference in the percentage of total CD4 and CD8 T tumor infiltrating cells between the groups (S2 Fig). Moreover, when analyzing subpopulations of CD4 T cells there was a higher percentage (p<0.05) of CM cells on Hybrid group and of EM cells on *P*. *acnes* group in relation to the other groups (S2 Fig). On the other hand, there was only higher percentage (p<0.05) of naïve CD8 T cells on *P*. *acnes* group when compared to all of the other groups (S2 Fig). Besides, there was an augment in the percentage (p<0.05) of activated CD4 and CD8 T infiltrating tumor cells in the Hybrid+*P*. *acnes* group in relation to PBS group (S2 Fig).

### The hybrid vaccine injected with *P*. *acnes* polarized the Th1 immune response to melanoma antigens

CD4 and CD8 T lymphocytes isolated from total splenocytes were obtained and cultured with tumor antigens to analyze the cytokine profile of tumor-specific T cells induced by vaccination.

**Fig 4:**
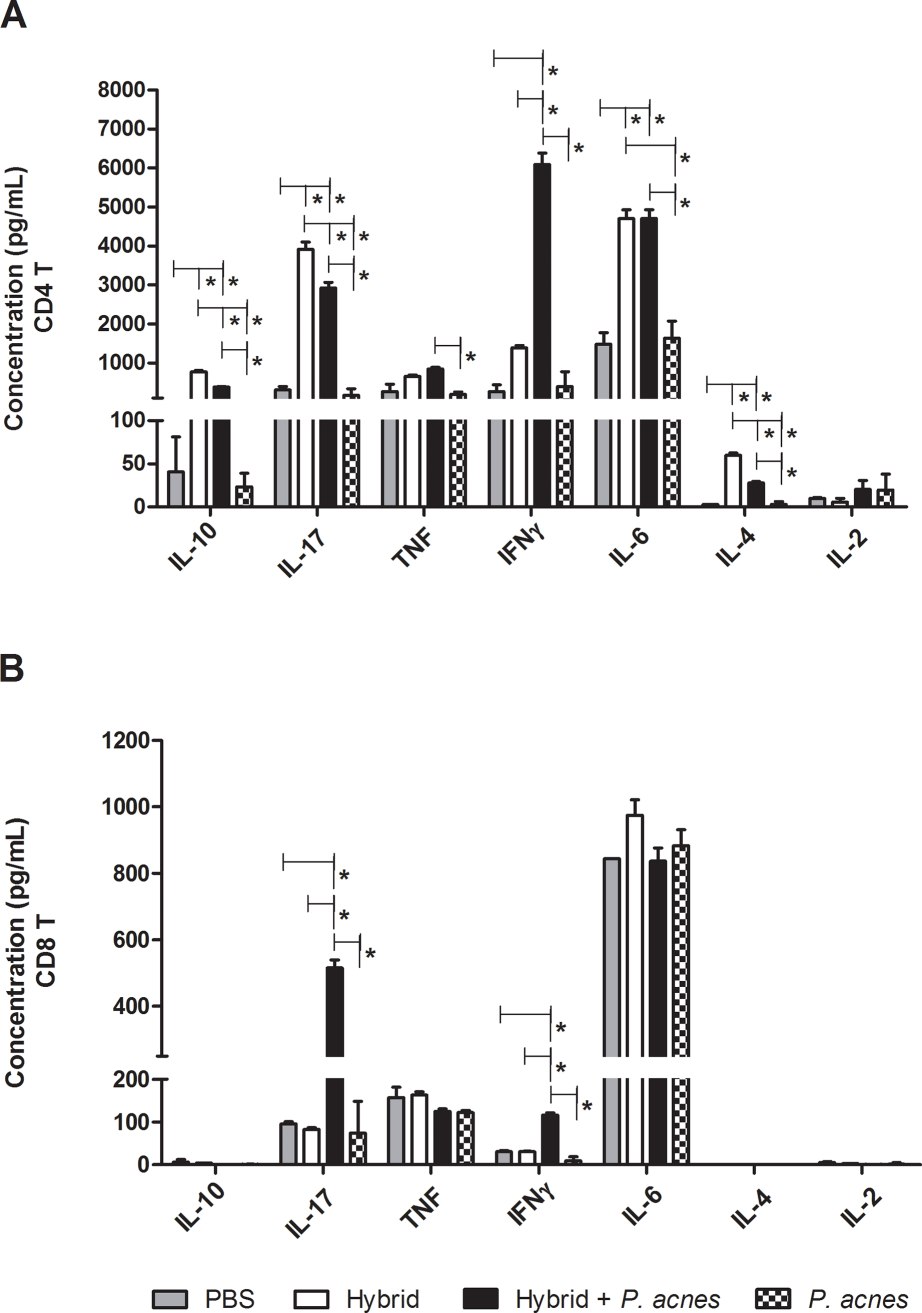
The addition of *P*. *acnes* to the hybrid vaccine polarized the CD4 T cell response to Th1 pattern and increased IFN-γ and IL-17 synthesis by CD8 T cells. Two weeks after the last immunization, CD4 T (A) and CD8 T (B) lymphocytes from C57Bl/6 mice (n = 3) were isolated from total spleen cells (BD FACSAria II-Cell Sorter) and cultured *ex vivo* with DCs and tumor antigens. The supernatants from these cultures were collected after 5 days, and the cytokine profiles were analyzed using a Cytometric Bead Array (BD) to detect IL-10, IL-17, TNF-α, IFN-γ, IL-6, IL-4 and IL-2 levels. The data (mean ± SEM) shown in the graphs are representative of two independent experiments. ANOVA with Tukey’s post-test *p<0.05, **p<0.01, ***p<0.001.

The addition of *P*. *acnes* to the hybrid vaccine increased IFN-γ production and reduced IL-10 and IL-4 synthesis by CD4 T cells compared to the Hybrid group, polarizing the immune response to a Th1 pattern (Fig 4A).

Higher concentrations of IL-17 and IFN-γ were observed in the supernatant of CD8 T lymphocytes from the Hybrid+*P*. *acnes* group compared to cultures from the PBS, Hybrid and *P*. *acnes* groups (Fig 4B).

### Improvement of spleen cell cytotoxicity toward murine melanoma cells

We investigated the ability of spleen cells from the immunized groups to induce the lysis of B16F10 cells. Splenocytes were obtained 24 hours after the last immunization and cultured with B16F10 cells that had previously been incubated with [methyl-^3^H] thymidine.

**Fig 5:**
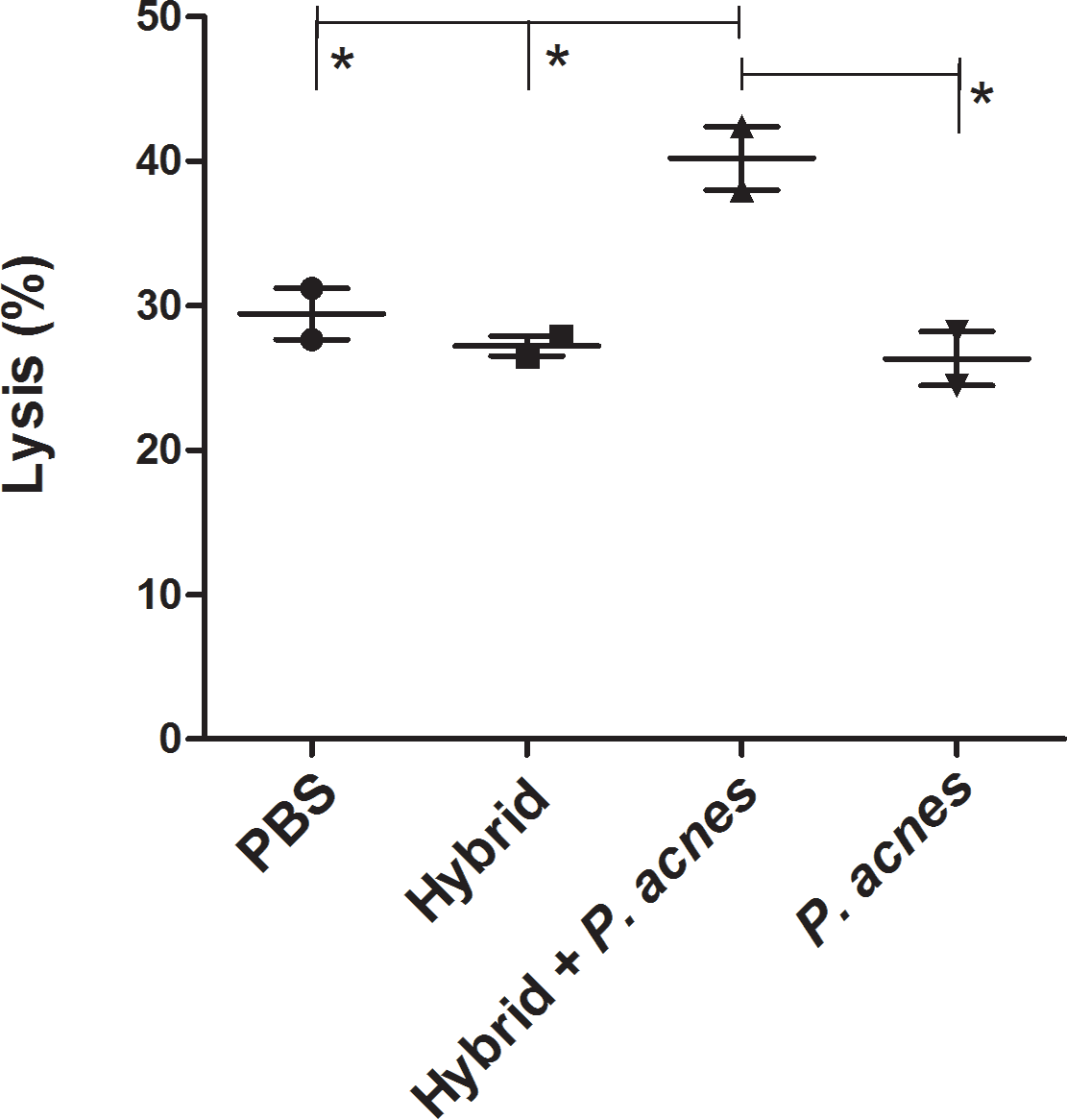
The addition of *P*. *acnes* to the hybrid vaccine increased cytotoxic activity of splenocytes toward B16F10 cells. Twenty-four hours after the last immunization of C57Bl/6 mice (n = 3), spleen cells were obtained and cultured with B16F10 cells that had previously been incubated with [methyl-^3^H] thymidine. The cells were maintained at a proportion of 1 target cell to 50 effector cells for 3.5 hours (37°C, 5% CO_2_). The cells were harvested and radioactivity was measured using a β-counter and converted to a lysis percentage. The data shown in the graph represent the mean ± SEM of two independent experiments. ANOVA with Tukey’s post-test * p<0.05.

Cells from the Hybrid+*P*. *acnes* group were more cytotoxic than cells from the other groups, and the percentage of B16F10 cells lysed by this group was 40.2±2.2%, whereas the percentages for the other groups were 27.2±0.7% (Hybrid), 29.4±1.8% (PBS) and 26.3±1.9% (*P*. *acnes*) (Fig 5).

### The addition of *P*. *acnes* to the hybrid vaccine reduced tumor progression

**Fig 6:**
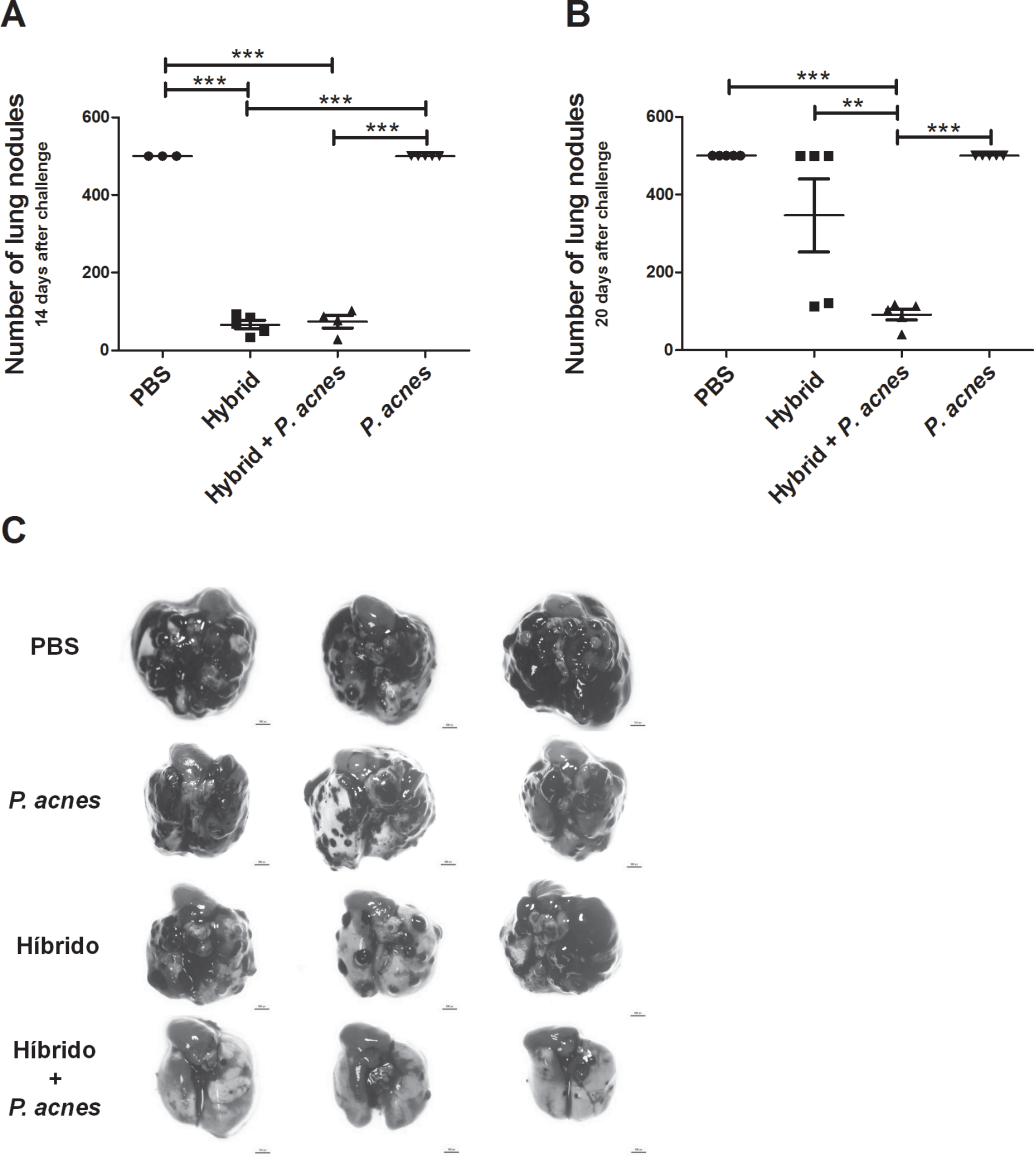
The addition of *P*. *acnes* to the hybrid vaccine prevented tumor progression. C57Bl/6 mice (n = 5) were sc immunized with the hybrid vaccine to which *P*. *acnes* had or had not been added on days 0 and 14. Mice from the control groups received PBS or *P*. *acnes* only. On day 21, the animals were intravenously challenged with 3x10^5^ B16F10 cells. The number of lung nodules was determined on days 35 (A) or 41 (B). Images of the lungs were obtained on day 41 (C). The images and data (mean ± SEM) shown in the graphs are representative of two independent experiments. Magnification 10x. Length of the scale bar = 100 px. ANOVA with Tukey’s post-test **p<0.01, ***p<0.001.

Next, we verified the ability of these vaccines to induce protection against pulmonary colonization by B16F10 cells *in vivo*. Seven days after the second immunization, the animals were intravenously challenged with B16F10 cells, and the number of lung nodules was determined after 14 (Fig 6A) or 20 (Fig 6B) days.

Fourteen days after challenge, the groups treated with the hybrid vaccine with or without *P*. *acnes* were equally protected against pulmonary colonization (mean numbers of lung nodules = 73.5 and 66.2, respectively) compared to the PBS and *P*. *acnes* groups (mean number of lung nodules in both group = 500) (Fig 6A). However, at twenty days after challenge, only the Hybrid+*P*. *acnes* group displayed a reduced number of lung nodules (91.4 nodules) compared to the Hybrid (346.8 nodules), PBS (500 nodules) and *P*. *acnes* (500 nodules) groups (Fig 6B and 6C). Thus, immunization with the hybrid vaccine and *P*. *acnes* uniquely prevented late tumor progression. Notably, at 20 days after challenge, the Hybrid+*P*. *acnes* group maintained a similar number of lung nodules to the number observed at 14 days after B16F10 cell inoculation (73.5 nodules) (Fig 6A and 6B). However, the Hybrid group progressed from 66.2 nodules to 346.8 nodules, and, as expected, the *P*. *acnes* and PBS groups continued to exhibit intense pulmonary colonization (Fig 6A and 6B).

Indeed, images of the lungs clearly revealed the differences in late tumor colonization between these groups. Almost no B16F10 nodules were observed in the Hybrid+*P*. *acnes* group, and the existing nodules were much smaller than the nodules observed in the other three groups (Fig 6C).

## Discussion

Hybrid vaccines comprising fused DCs and tumor cells have produced interesting results in preclinical and clinical trials. In experimental models, hybrid vaccines induce the production of cytotoxic T lymphocytes and prevent the growth of pre-implanted tumor cells [35, 36]. According to clinical studies, hybrid vaccines are nontoxic and capable of inducing immunological and clinical antitumor responses [6, 7]. However, the clinical response is not as vigorous as the responses observed in animal models, indicating a need to improve the response triggered. Some studies have evaluated the effect of hybrid vaccines and the third signal required to induce a robust immune response, such as genetic modifications in tumor cells to induce IL-12 secretion [37, 38]. Another strategy to provide this signal would be to add an adjuvant, particularly a biological adjuvant, such as bacteria or bacterial products. In the past decade, we have studied the adjuvant effect of *P*. *acnes* on different murine models due its ability to modulate several steps of innate and specific immune responses [18, 26–28, 31, 32, 39, 40].

Here, we verified that the addition of *P*. *acnes* to the hybrid vaccine improved specific humoral and cellular immune responses to melanoma. The killed *P*. *acnes* suspension not only increased the total IgG, IgG1, IgG2b and IgG2c titers specific to B16F10 antigens (Fig 1) but also polarized the antibody response to a Th1 pattern (Table 1), which was protective in this model [41]. Similar results were shown in our previous study. The addition of *P*. *acnes* to a plasmid DNA vaccine against *Trypanosoma cruzi* expressing the trans-sialidase as a transgene, which increased specific IgG2a titers in BALB/c mice, also promoted the polarization of the antibody response to a Th1 pattern [32].

The specific antibodies against B16F10 can participate of mechanisms that mediate tumor cell killing such as complement-dependent cytotoxicity and antibody-dependent cellular cytotoxicity (ADCC) [42]. In a melanoma mice model, Nimmerjahn & Ravetch (2005) compared the ability of different class-switched antibodies, specific for the melanosome gp75 antigen (TA99), to mediate tumor clearance. The authors observed that TA99 carrying IgG2a constant regions displayed enhanced ADCC in the metastatic melanoma model compared with these antibodies bearing IgG1 constant regions [43]. Thus, the polarization of the immune response to the Th1 profile and the increase of IgG2c titer (allele expressed by C57Bl/6 equivalent to IgG2a) in the Hybrid+*P*. *acnes* group reinforce the importance of the association of this adjuvant to the hybrid vaccine, since this modulation of the immune response may confer enhanced protection against B16F10.

The Th1 pattern induced by immunization with the hybrid vaccine and *P*. *acnes* was also revealed by the cytokine profiles of tumor-specific T cells stimulated *in vitro* with tumor antigens. Hybrid vaccine increased the production of IL-4 and IL-17 by CD4 T cells in relation to PBS group e did not alter their IFN-γ synthesis. However, *P*. *acnes* association to hybrid vaccine induced high levels of IFN-γ production and reduced IL-4 and IL-17 synthesis by CD4 T cells in relation to Hybrid group (Fig 4A). While Th17 cells are prevalent in Hybrid group, despite the Th1 cells seems to be the major subset in Hybrid+*P*. *acnes* group, we also detected a Th17 population.

Kryczek *et al*. (2007) described the presence of Th17 cells on tumoral microenvironment in mice and humans [44], but the role of Th17 cells on tumor immunity is controversial yet. Pro-inflammatory cytokines synthesized by Th17 cells, such as IL-17 and IL-23, has been associated to tumor neovascularization and reduced CD8 T cell infiltration into the transformed tissue [45, 46]. Nevertheless, Muranski *et al*. (2008) observed that Th17-polarized cells mediated destruction of advanced melanoma after being adoptively transferred to tumor-bearing mice [47]. Moreover, it was observed increased Th17 and Th1 cell populations in melanoma patients, which had immunologic response against melanoma antigens after treatment with dendritic cell loaded with B16 cell lysate, and this effect was associated with a prolonged patient survival [48]. Thus, further studies are needed to clarify the role of these subpopulations of helper T cells, however, in the immune response against melanoma there seems to be a cooperation between Th1 and Th17 cells.

In our previous study, *P*. *acnes* improved Th1-specific immune response to a plasmid DNA *T*. *cruzi* vaccine reducing parasitemia compared to the control group [32]. In addition, *P*. *acnes* potentiates or suppresses the Th2 response to OVA in a murine asthma model, depending on the *P*. *acnes* treatment protocol [26, 27]. The modulation of the immune response by this bacterium reflects a direct action on APCs, in which *P*. *acnes* induces the activation of innate immune cells via TLR2 and TLR9 signaling [49, 50] and modifies the expression of costimulatory molecules and TLRs on immune cells [28, 31, 39, 40].

In the present study, we also detected the *in vivo* expansion of T cells after immunization with the hybrid vaccine, which increased the absolute numbers of total CD4 T and CD8 T cells compared to the PBS group (Fig 2). This vaccine induced the production of EM CD4 T cells and CM CD4 T and CD8 T cells (Fig 2). The addition of *P*. *acnes* to the hybrid vaccine amplified the proliferation and differentiation of these cells *in vivo*, increasing the absolute number and proportion of CM and EM CD4 and CD8 T cells compared to the Hybrid, PBS and *P*. *acnes* groups (Figs 2 and 3). Although this phenotype had been performed in the total cells, it was observed a higher number of CM and EM CD4 and CD8 T cells in Hybrid+*P*. *acnes* group when compared to *P*. *acnes* group, which allows infer that there was an augment in the number of CM and EM antigen-specific T cells subpopulations. These results are consistent with the higher absolute numbers of activated T lymphocytes in the Hybrid+*P*. *acnes* group than in the Hybrid, PBS and *P*. *acnes* groups (Fig 2). Indeed, CD69 is one of the earliest cell surface antigens to be expressed by T cells after antigen recognition and acts as costimulatory signal for T cell proliferation [51]. Furthermore, the addition of *P*. *acnes* to the hybrid vaccine improved the induction of memory cell proliferation, which is important to the longevity of the antitumor immune response.

When CD8 T cells from the vaccinated or control groups were stimulated with tumor antigens *in vitro*, increased levels of IFN-γ and IL-17 were detected in the supernatants from cells from the Hybrid+*P*. *acnes* group compared to the PBS, Hybrid and *P*. *acnes* groups (Fig 4). The role of IFN-γ in antitumor immunity has previously been described, and this cytokine exerts antitumor effects by acting directly on tumor cells or indirectly, through immune system activation [52, 53]. IL-17-secreting CD8 T lymphocytes display enhanced antitumor activity after their conversion to IFNγ-producing cells through adoptive transfer in experimental models, mediating the regression of established tumors [54].

Based on our results is not possible know if each T cell from Hybrid+*P*. *acnes* group is producing a higher amount of cytokine or if these enhancement in concentration of different cytokines, such as TNF and IFN-γ, is a result of more cells secreting it. More experiments are needed to answer this question, but independently of how it occurs the environment created by these cytokines is very important to an antitumor immune response. However, recently Teixeira et al. (2018) demonstrated that *P*. *acnes* association to a DNA vaccine containing 18 CD4^+^ T cell epitopes from human immunodeficiency virus (HIV) increased the proliferation of HIV-1-specific CD4^+^ and CD8^+^ T lymphocytes, including a higher percentage of CD4 T cells producing IFN-γ and TNF-α [55].

Another mandatory immune response to eliminate tumor cells is the cytotoxic activity of immune cells; thus, we investigated this function in splenocytes from the treated groups *in vitro*. Splenocytes from the Hybrid+*P*. *acnes* group showed greater cytotoxic activity against B16F10 cells than the PBS, *P*. *acnes* and Hybrid groups (Fig 5). This effect might be related to all previous results, as the addition of *P*. *acnes* to the hybrid vaccine polarizes the immune response to a Th1 pattern and induces the expansion of CM and EM CD4 and CD8 T cells, thereby inducing tumor cell lysis. Moreover, T cells specific to melanoma antigens from Hybrid+*P*. *acnes* group produced higher levels of IFN-γ, which in turn activates macrophages, natural killer (NK) and NKT cells that also mediate tumor cell death. *P*. *acnes* has been shown to increase the tumoricidal activity of macrophages and NKT cells [17, 18]. We also verified that cells from the peritoneal exudate of *P*. *acnes*-treated mice are cytotoxic to melanoma cells, and one of the main mechanisms mediating the effect of this bacterium is nitric oxide release [18].

Interestingly, the protective effect of the combination of the hybrid vaccine and *P*. *acnes* was observed 20 days after challenge *in vivo*; however, tumor progression in the Hybrid was similar to the control group during the same period (Fig 6). Moreover, the combination of the hybrid vaccine and *P*. *acnes* reduced the number and size of lung nodules, as lung nodules from animals in the Hybrid+*P*. *acnes* group were much smaller than the nodules observed in the Hybrid group (Fig 6C). When memory phenotype of tumor infiltrating cells was analyzed, it was observed that 20 days after challenge there was no difference in the percentage of CD4 and CD8 T cells inside the melanoma colonized lungs. However, in the Hybrid and *P*. *acnes* groups in which similar pulmonary colonization to the PBS group were observed, there was an increase in the percentage of CM and EM CD4 T cells, respectively, in relation to the Hybrid+*P*. *acnes* group (S2 Fig). It is possible that at the time in which we obtained tumor infiltrating cells (20 days after challenge), where fewer and smaller nodules were present at Hybrid+*P*. *acnes* group, it had already decreased the frequency of memory cells once they had a better resolution of tumor compared to PBS, Hybrid and *P*. *acnes* groups. However, further investigations are needed to characterize tumor infiltrating cells and determine the main mechanisms responsible for tumor growth inhibition, once the activity of these cells maybe could better explain our results that the proportion of them inside the tumor. Tsuda et al. (2011) demonstrated that intratumoral injection of *P*. *acnes* protected against skin melanoma progression *in vivo* and observed infiltrating T cells expressing TNF-α and IFN-γ at tumor lesions [56].

Hybrid vaccines have been shown to prevent tumor growth [35, 36]. Nevertheless, in this study, we reported an important adjuvant effect of the addition of *P*. *acnes* to the hybrid vaccine on prolonging the inhibition of tumor growth, likely reflecting the increased production of T memory cells and their ability to kill tumor cells. The expansion of T memory cells was detected 24 hours after the second immunization with the hybrid vaccine and *P*. *acnes*, and this effect was maintained for 28 more days, when the last evaluation of tumor growth inhibition was conducted *in vivo*.

In summary, *P*. *acnes* enhances the immunogenicity of the hybrid vaccine by improving the specific immune response elicited by this vaccine and prolonging its control of tumor growth. Moreover, this study presents an interesting approach that deserves further investigation for potential clinical applications.

## Supporting information

S1 Checklist Animal research: Reporting of *In Vivo* experiments. (PDF)

S1 Fig Representation of the gating strategy used to determine the T cell memory phenotypes. Cells were gated in an FSCxSSC dot plot (A), and doublets were excluded (B). CD4 (C) or CD8 (D) T cells were selected and analyzed for the concomitant expression of CD62L and CD44 (E and F) or CD69 expression (G and H) to determine the percentages of *naïve* (CD44^low^CD62L^high^), EM (CD44^high^CD62L^low^) and CM (CD44^high^CD62L^high^) CD4 and CD8 T cells and degree of activation.
(TIF)

S2 Fig Memory phenotype of tumor infiltrating CD4 and CD8 T lymphocytes. Seven days after the second vaccine dose, C57Bl/6 mice (n = 3) were challenge with B16F10 intravenously. Twenty days later, animals were euthanized, lung were extracted and from this tissue was obtained a cell suspension. Tumor infiltrating lymphocytes were enriched using Percoll gradient. Subsequently, the cells were stained with fluorochrome-conjugated monoclonal antibodies and analyzed using flow cytometry. The mean ± SEM percentage of CD4 and CD8 T cells (CD3^+^CD4^+^ and CD3^+^CD8^+^) (A) and subpopulations of *naïve* (CD44^low^CD62L^high^) (B), CM (CD44^high^CD62L^high^) (C) and EM cells (CD44^high^CD62L^low^) (D) are presented in the graphs. The percentage of activated CD4 and CD8 T cells (CD69^+^) (E) and their degree of activation based on CD69 mean fluorescence intensity (MFI) (F) were also investigated. ANOVA with Tukey’s post-test *p<0.05, **p<0.01.
(TIF)
