# Supplementary figures and images for: Killed Propionibacterium acnes enhances immunogenicity and tumor growth control of a dendritic-tumor cell hybrid vaccine in a murine melanoma model
Source: PLoS One. 2018 Oct 9;13(10):e0205148. doi: 10.1371/journal.pone.0205148 (PMC6177168; doi:10.1371/journal.pone.0205148)

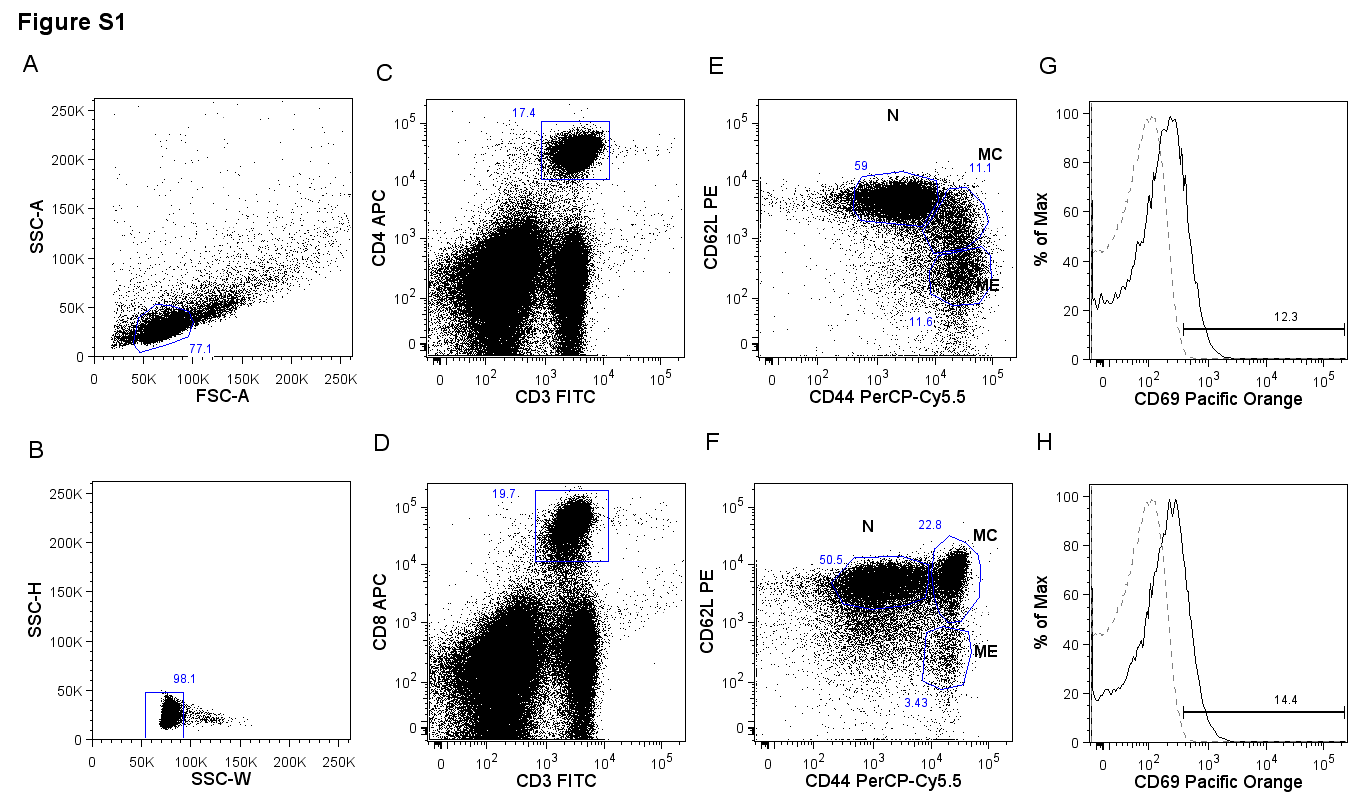

Supplement: S1 Fig — Cells were gated in an FSCxSSC dot plot (A), and doublets were excluded (B). CD4 (C) or CD8 (D) T cells were selected and analyzed for the concomitant expression of CD62L and CD44 (E and F) or CD69 expression (G and H) to determine the percentages of naïve (CD44lowCD62Lhigh), EM (CD44highCD62Llow) and CM (CD44highCD62Lhigh) CD4 and CD8 T cells and degree of activation. (TIF) [file pone.0205148.s002.tif]

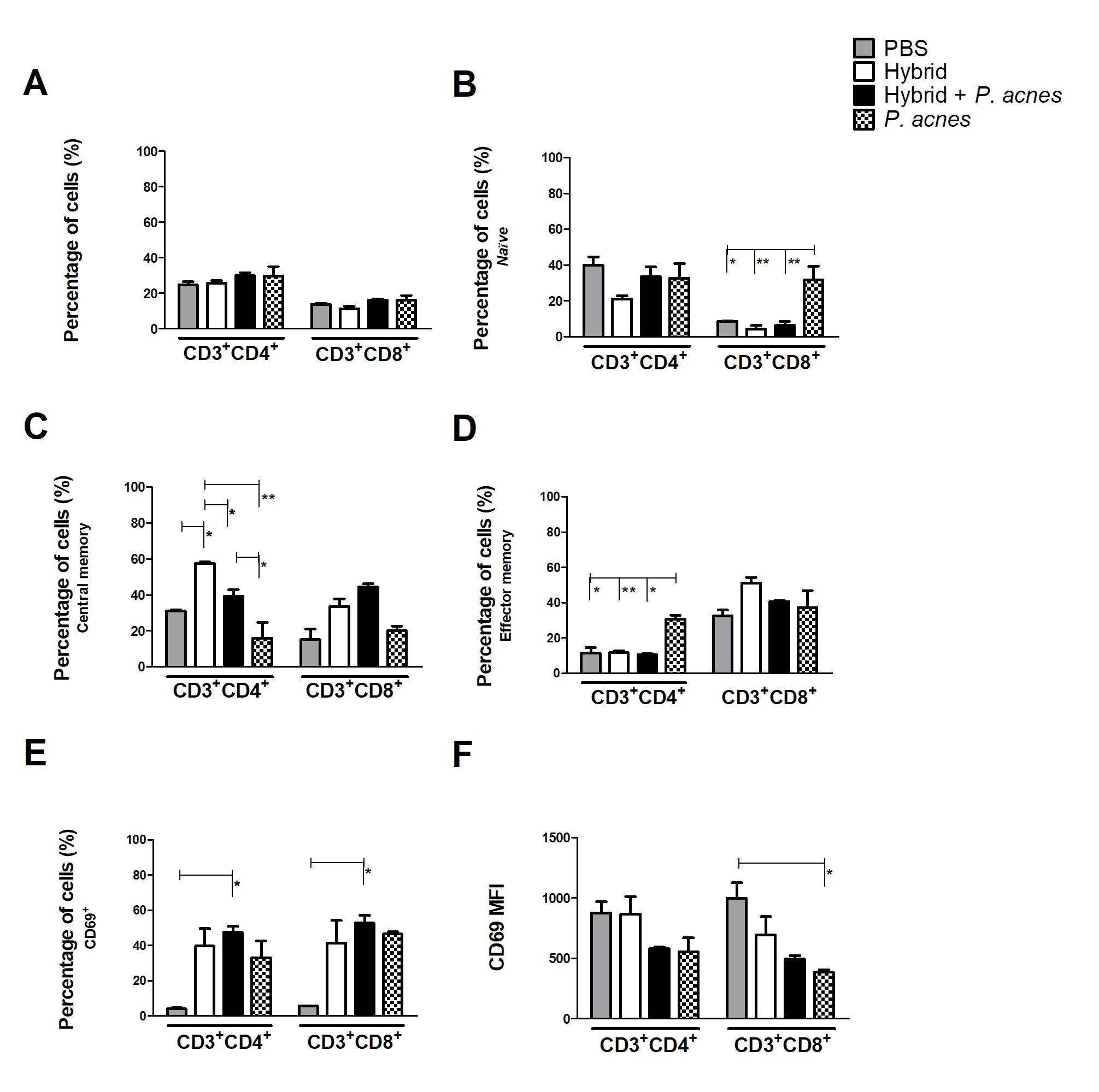

Supplement: S2 Fig — Seven days after the second vaccine dose, C57Bl/6 mice (n = 3) were challenge with B16F10 intravenously. Twenty days later, animals were euthanized, lung were extracted and from this tissue was obtained a cell suspension. Tumor infiltrating lymphocytes were enriched using Percoll gradient. Subsequently, the cells were stained with fluorochrome-conjugated monoclonal antibodies and analyzed using flow cytometry. The mean ± SEM percentage of CD4 and CD8 T cells (CD3+CD4+ and CD3+CD8+) (A) and subpopulations of naïve (CD44lowCD62Lhigh) (B), CM (CD44highCD62Lhigh) (C) and EM cells (CD44highCD62Llow) (D) are presented in the graphs. The percentage of activated CD4 and CD8 T cells (CD69+) (E) and their degree of activation based on CD69 mean fluorescence intensity (MFI) (F) were also investigated. ANOVA with Tukey’s post-test *p<0.05, **p<0.01. (TIF) [file pone.0205148.s003.tif]
